# Can high-sensitivity C-reactive protein be a routine trans-diagnostic biomarker for thoughts of death and suicidal attempts?

**DOI:** 10.1192/j.eurpsy.2024.1631

**Published:** 2024-08-27

**Authors:** L. Cavallo, L. Orsolini, U. Volpe

**Affiliations:** Unit of Clinical Psychiatry, Department of Neurosciences/DIMSC, Polytechnic University of Marche, Ancona, Italy

## Abstract

**Introduction:**

Several studies have shown an association between suicidal behavior and increased C-reactive-protein (CRP) levels (Ghayour-Mobarhan M. *et al*. Comb Chem High Throughput Screen 2022; 25 1047-1057) although most studies evaluated the association between CRP levels and suicidal ideation in depressed patients (Olié E. *et al*. Eur Neuropsychopharmacol 2015; 25 1824-31).

**Objectives:**

Our study assessed baseline high-sensitivity CRP (hsCRP) levels in a cohort of adult inpatients affected by severe mental illness (SMI) and their association with Mini-International Neuropsychiatric Interview-5 subscale suicidality (MINI-5-s).

**Methods:**

A naturalistic, observational, cross-sectional study was carried out by retrospectively recruiting 127 adult SMI inpatients, excluding patients with an organic pathology. HsCRP levels were assessed at the ward admission. To assess the suicidal behaviour all patients filled the same day the MINI-5-s.

**Results:**

The number of patients with hsCRP>3mg/l were significantly higher among those with thoughts of death (p=0.002) and suicidal attempt (p=0.026). No statistically significant associations were observed between hsCRP levels and other suicidality dimensions.

Limitations: Small sample size, heterogeneous diagnoses, lack of diagnostic sub-analysis, cross-sectional design, and lack of a healthy control group.

**Conclusions:**

The study reveals a transdiagnostic association between inflammation, thoughts of death and suicidal attempt in SMI inpatients. Our preliminary findings could support a routine introduction of hsCRP measurement, due to its relatively low cost, possible utility in trans- diagnostically suicide risk assessment. Large-scale clinical trials would be recommended to evaluate the effects of early anti-inflammatory therapy in patients with death ideation and/or suicidal attempt and concomitant low-grade hsCRP elevation. HsCRP could potentially represent an early biomarker for suicidal risk.

**Disclosure of Interest:**

None Declared

